# Impact of Transcranial Direct Current Stimulation in Pain, Fatigue, and Health Quality of Life of Patients with Idiopathic Inflammatory Myopathies: A Randomized, Double-Blind, Sham-Controlled Crossover Clinical Trial

**DOI:** 10.1155/2024/1583506

**Published:** 2024-02-01

**Authors:** Rafael Giovani Missé, Alexandre Moura dos Santos, Isabela Bruna Pires Borges, Marcus Vinicius Grecco, Marlise Sítima Mendes Simões Faria, Lorenza Rosa Silvério da Silva, Bruna Lindoso Correia, Ana Woo Sook Kim, Clarice Tanaka, Júlia Maria D'Andrea Greve, Abrahão Fontes Baptista, Samuel Katsuyuki Shinjo

**Affiliations:** ^1^Division of Rheumatology, Faculdade de Medicina FMUSP, Universidade de Sao Paulo, Sao Paulo, Brazil; ^2^Laboratório do Estudo do Movimento, Instituto de Ortopedia e Traumatologia (IOT), Hospital das Clinicas HCFMUSP, Faculdade de Medicina, Universidade de Sao Paulo, Sao Paulo, SP, Brazil; ^3^Núcleo de Assistência e Pesquisa em Neuromodulação (NAPeN), Sao Paulo, SP, Brazil; ^4^Departamento de Fisioterapia, Fonoaudiologia e Terapia Ocupacional, Hospital das Clínicas HCFMUSP, Faculdade de Medicina, Universidade de Sao Paulo, Sao Paulo, SP, Brazil; ^5^Center for Mathematics, Computation and Cognition, Federal University of ABC (UFABC), São Bernardo do Campo, SP, Brazil

## Abstract

**Objectives:**

To assess the effectiveness of transcranial direct current stimulation (tDCS) for pain, fatigue, physical function, and health-related quality of life in patients with idiopathic inflammatory myopathy (IIM).

**Methods:**

This randomized, double-blind, sham-controlled, crossover clinical trial enrolled IIM patients with fatigue and pain who received tDCS (20 min, 2 mA) or sham stimulation for 10 daily sessions. Electrodes were placed according to the 10/20 EEG system. Both the groups underwent aerobic exercise training during the intervention period. The patients were evaluated for disease perception, pain, and fatigue using uni-multidimensional questionnaires and physical tests in the periods before and after the first and second interventions and after 12 weeks of follow-up.

**Results:**

After the tDCS intervention, a reduction in the general score of multidimensional pain of 32.0 (1.5-38.0) *vs.* 0.0 (0.0-13.4) with effect size (ES) of -0.78 was noted, and after sham intervention, a reduction of 26.0 (0.0-37.0) *vs*. 5.0 (0.0-19.2) with ES of -0.54 (*P* = 0.047) was also noted. Similar results were evidenced with fatigue (22.5 (15.4-33.2) *vs.* 5.5 (0.0-14.6) with ES of -0.82) and sham intervention (21.0 (15.8-29.5) *vs.* 4.0 (4.0-17.5) with ES of -0.80 (*P* = 0.012)). There were no differences in the domains of the fatigue and pain questionnaires. Adherence was observed in 88.8% of the patients without adverse events.

**Conclusion:**

The association of tDCS with aerobic training promoted additional effects in relation to the group subjected to placebo stimulation on general pain and fatigue scores, as well as on pain intensity, without changes in the subdomains of the pain and fatigue questionnaire. This trial is registered with NCT04678635.

## 1. Introduction

Idiopathic inflammatory myopathies (IIMs) or systemic autoimmune myopathies are a group of rare autoimmune rheumatic diseases mainly characterized by skeletal muscle involvement, which results in the loss of physical function and health-related quality of life (HQoL) [1, 2]. Based on demographic, clinical, laboratory, and histopathological data, IIMs are classified as polymyositis (PM), dermatomyositis (DM), antisynthetase syndrome (ASSD), and immune-mediated necrotizing myopathy (IMNM) [1–3].

Pain and fatigue have been associated with job loss, disability, and loss of quality of life among several autoimmune rheumatic diseases including IIMs [4, 5]. In this context, strategies to improve these parameters are fundamentally relevant.

Exercise training is the most recommended strategy to improve physical function and HQoL in patients with IIMs. Additionally, exercise training has been associated with poor pain and fatigue in these patients [6, 7]. Transcranial direct current stimulation (tDCS) is a noninvasive brain stimulation technique that uses low-intensity electrical stimulation delivered to the brain. tDCS has been used for pain relief, fatigue relief, and physical and cognitive improvement in several chronic diseases [8].

Nonetheless, few studies have supported the use of tDCS for rheumatic diseases. However, relevant advances have been made in patients with fibromyalgia and Sjögren's syndrome [9–11].

A recent study conducted by Pinto et al. [11] demonstrated that tDCS applied over the dorsolateral prefrontal cortex (DPLC) without association with other therapies was effective in improving sleep quality and physical function in patients with Sjögren's syndrome [11].

In this context, a clinical trial by Mendonca et al. [10] showed that tDCS on the primary motor cortex associated with moderate aerobic exercise training had an additive effect on the perception of pain in patients with fibromyalgia compared to tDCS without other interventions. The authors attributed these results to a potential neural facilitation effect through the combination of tDCS and aerobic exercise training stimuli [10].

In patients with IIMs, a recent study by our group demonstrated that three sessions of tDCS without an association with another nonpharmacological therapeutic approach were safe and improved physical function [12]. Encouraging results suggest a potential additive effect of tDCS associated with another nonpharmacological approach for these diseases [12]. Nonetheless, there is still no evidence regarding the potential analgesic effect of tDCS in patients with IIMs, and no data are available regarding the effect of longer intervention duration and potential additive effect on the association with aerobic exercise training. Therefore, this study is aimed at assessing the safety and efficacy of these parameters. We considered chronic fatigue as the primary outcome, which was assessed using the Fatigue Severity Scale (FSS). Additionally, we evaluated the effect on the outcome by comparing the pre- and postintervention measurements.

## 2. Materials and Methods

### 2.1. Study Design

This single-center, randomized, double-blind, sham-controlled crossover clinical trial was conducted between September 2019 and March 2022. This study was approved by the local ethics committee (CAAE: 41916820.3000.0068) and was registered on the ClinicalTrials.gov portal (NCT04678635). A total of 200 patients with IIMs were regularly followed up at our tertiary outpatient rheumatology clinic.

Patients with classification for dermatomyositis (DM), clinically amyopathic dermatomyositis (CADM) based on the classification criteria of the European League Against Rheumatism/American College of Rheumatology (EULAR/ACR) 2017 [2], and immune-mediated necrotizing myopathies (IMNM), according to the classification criteria of Allenbach et al. [13], were included. The antisynthetase syndrome (ASSD) was based on the definition of Behrens Pinto et al. [14].

### 2.2. Inclusion Criteria

According to the International Myositis Assessment and Clinical Studies Group (IMACS) score, patients were aged >18 years, with chronic pain and fatigue, disease in remission, or minimal disease activity.

### 2.3. Exclusion Criteria

Patients with neoplasms, users of cardiac pacemakers, users of clips or metallic cranial prostheses, pregnant women, history of seizures or epilepsy, users of centrally acting drugs or drugs that lower the seizure threshold, and skin lesions (scalp in electrode application) were the exclusion criteria.

Before beginning the study, all patients were informed of the study procedures and signed an informed consent form.

### 2.4. Study Flowchart

Patients were screened at the outpatient rheumatology clinic for pain and fatigue perception and were subsequently enrolled in a second interview to collect clinical, demographic, and laboratory data, pain, fatigue, muscle function, and resistance. The patients were randomized by health professionals external to the research group in a 1 : 1 ratio concerning the clinical characteristics, fatigue, and pain levels assessed by unidimensional questionnaires, such as the visual analog scale for fatigue (VASf), FSS, and visual analog scale for pain (VASp). Patients were assessed at four time points: baseline, after the intervention, postwashout at 12 weeks, and postsecond interventions (Supplementary Figure S1).

### 2.5. Assessments

Data were collected before and after tDCS and associated with aerobic exercise intervention.

- Demographics: age and sex
- Anthropometric: weight, height, and body mass index (BMI)
- Lifestyle: smoking, alcohol consumption, and physical activity
- Comorbidities: dyslipidemia (total plasma cholesterol > 200 mg/dL, HDL − cholesterol < 40 mg/dL (men) or<50 mg/dL (women), LDL − cholesterol > 130 mg/dL, triglycerides > 150 mg/dL, or in pharmacological treatment for dyslipidemia), systemic arterial hypertension, diabetes mellitus, and depressive symptoms
- Pharmacological treatment (previous and current): glucocorticoids (immunosuppressants, immunomodulators, and/or immunobiological), analgesics, antidepressants, and statins
- Disease status: Manual Muscle Testing- (MMT-) 8, Myositis Disease Activity Assessment Visual Analog Scales (MYOACT), global assessment of the disease by physicians and patients through EVA, and Health Assessment Questionnaire (HAQ) [15, 16]
- The laboratory test results included creatine phosphokinase (CPK), aspartate aminotransferase (AST), and alanine aminotransferase (AST) levels
- HQoL: short-form questionnaire (SF-36) [17]
- Physical function: Short Physical Performance Battery (SPPB) [18], sit-to-stand (STS) test [19], and timed up and go (TUG) test [20]
- Depressive symptoms: the Beck Depression Inventory (BDI) is a self-assessment scale validated in Brazil [21]
- Anxiety symptoms: the State-Trait Anxiety Inventory (STAI) is a self-completed scale validated for the Brazilian population. The questionnaire assesses nonspecific aspects of anxiety that may be present in stressful situations and some cognitive components of anxiety [22]
- Sleep quality: Pittsburgh scale questionnaire [23]
- The overall pain assessment included the short-form McGill Pain Questionnaire 2 (SF-MPQ-2) [24] and the Fatigue Severity Scale (FSS) [25]

## 3. Procedures

### 3.1. Randomization

This was performed using the “randomizeR” package in the R statistical environment. We employed simple randomization without any restrictions such as blocking or block size. To maintain blinding, the allocation sequence was generated by a researcher who was not involved in the study, participant recruitment, or allocation. Researchers involved in data collection and statistical analysis were blinded to the treatment allocation until the data collection was completed. Additionally, the device was programmed to generate an electrical current similar to that applied in the tDCS group for 30 s and was then gradually reduced to simulate the intervention. These practices were implemented to uphold the integrity of the study, minimize bias, and ensure proper randomization while preserving the confidentiality of the sequence.

### 3.2. tDCS

The anode energy of tDCS was sourced from a battery-powered direct current generator (Activadose II, USA) exerted by two electrodes measuring 5 × 7 cm (35 cm^2^) (Ibramed, Brazil), covered by a vegetable sponge with saline solution, and fixed to the head of patients through orthopedic bands. The electrodes were placed on the primary motor cortex according to the International 10/20 system. The negatively charged electrode (cathode) was positioned at C3 or C4 (contralateral to the dominant limb), and the positively charged electrode (anode) was positioned in the supraorbital region ipsilateral to the dominant limb. The tDCS active current was applied at an electric current intensity of 2 mA and density of 0.057 mA/cm^2^ for 20 min, with a rise and fall ramp of 10 s. Placebo tDCS was applied with the same parameters, but the stimulus duration was only 30 s, according to previous studies that showed that this period was sufficient for the patient to perceive the presence of an electrical stimulus, but without a cerebral effect. During the session, the patients performed aerobic exercises. The 30 min interval between the session and the first assessment was chosen because it has already been shown to be an adequate period for the assessment of stimulation. tDCS was performed by a health professional and rheumatologist with adequate training to perform the procedure. The protocol, with a placebo or active stimulus, was performed by external health professionals.

### 3.3. Aerobic Exercise Training

The session consisted of walking on a treadmill for 30 min, and the intensity was verified using subjective perception of effort (RPE).

### 3.4. Adverse Events and Blinding

Adverse effects were evaluated during and after each session using a questionnaire on uncomfortable sensations such as burning, tingling, itching, burning (head), headache, nausea, fatigue, emotional lability, difficulty concentrating, and nervousness. After completion of the sessions, patients were asked about their respective allocations.

### 3.5. Adhesion

Adhesion to the protocol was recorded by the study coordinator and health professionals involved in this study using attendance sheets for each session.

### 3.6. Statistical Analysis

The normality of the data was tested using the Shapiro-Wilk test, and the homogeneity of variance was tested using the Levene test. Data are presented as mean ± standard deviation or median (interquartile range, 25^th^-75^th^) for continuous variables, and number (%) for categorical variables.

We calculated the sample size for the primary outcome, chronic fatigue. Therefore, for the two-tailed Wilcoxon-Mann–Whitney test, we considered an alpha error probability of 0.05 and a power of 0.8, to detect a moderate effect size of 0.5. The allocation was 1/1, and both groups were required to have a sample of 64 individuals.

To analyze the effects of tDCS on pain and fatigue parameters per session, individual percentage changes pre- and post-tDCS were calculated for each tDCS session as (post‐pre/pre) × 100. The same statistical treatment model was used for the global functionality data. The Student's *t*-test was used for the parametric data and the Mann–Whitney *U* test for nonparametric data. The effect size (ES) was calculated by considering the sample size and dispersion for performing the Hedge or Cohen tests. Analyses of clinical outcomes, physical assessment, perception, and the impact of fatigue were performed before and after the intervention using the Wilcoxon rank test. The significance level was set at *P* < 0.05. All data were analyzed using the RStudio software.

## 4. Results

A total of 85 patients completed the questionnaires on fatigue and pain perception. A total of 68 patients were removed: two due to disease activity, three due to other reasons, 53 due to work, and 10 refused to participate in the present study. Therefore, 17 patients underwent an aerobic exercise training program combined with tDCS or a sham intervention (Supplementary Figure S2).

Analyses were conducted on an intention-to-treat basis. However, it is important to note that there was an initial imbalance between tDCS (*n* = 16) and sham (*n* = 15) groups. Moreover, the data were reanalyzed using ANCOVA, considering metabolic equivalents (METs) as a covariate, as it was the only variable that showed a significant difference preintervention. Additionally, we examined the effect size (ES), enabling a clearer analysis of our findings.

Regarding demographic data, physical activity levels, comorbidities, and pharmacological treatment, no differences were observed before and after 12 weeks (Table 1).

### 4.1. Clinical Parameters

There was evidence of a significant difference between cervical flexion exercises in the Functional Index-3 for IIMs, results related to the intervention, and effects related to time. Similar findings were observed for HAQ and MMT-8, with the main effect characterized by the time effect. There was evidence of an interaction between intervention and time in Functional Index-3 for IIMs in cervical flexion and hip flexion exercises (Table 2).

### 4.2. Overall Physical Function

These differences were attributed to the intervention in the total SPPB test scores. In the SPPB strength domain, there was evidence of an interaction between the intervention and time. No differences were observed in the domains related to mobility and balance, as well as in the functional tests (Table 3).

### 4.3. HQoL

There was an interaction between the intervention and time in the domains of functional capacity and vitality. In the other domains of the SF-36, there were no differences between the groups (Table 4).

### 4.4. Perception and Severity of Fatigue

There was an interaction between intervention and time in the data regarding fatigue severity and the general perception of fatigue (Table 5).

### 4.5. Perception, Impact, and Severity of Pain

For pain variables, there were differences in the total scores on the McGill questionnaire. However, there were differences in the VAS scores for mean pain and time, similar to the effect of pain on disposition. Regarding the interactions between group and time, differences were observed in the VAS scores of average and maximum pain as well as in the impact of pain on general activity, ability to walk, work, and pleasure of life (Table 6).

### 4.6. Adverse Events and Blinding

Four (50.0%) patients undergoing tDCS+AE reported adverse events: two (25.0%) reported burning and two (25.0%) reported fatigue. In the Sham+AE group, two (22.2%) patients had an adverse event: one (11.1%) had symptoms of burning and one (11.1%) had symptoms of fatigue. Regarding blinding, five (62.6%) patients in the tDCS+AE group believed that they had received the active intervention, while eight (88.8%) patients in the Sham+AE group believed that they had received the intervention (Supplementary Table S1).

### 4.7. Adherence

There was 88.8% adherence to the protocol.

## 5. Discussion

This study is the first randomized clinical trial to assess the impact of tDCS with aerobic exercise training on pain and fatigue in patients with IIMs using uni-multidimensional validated questionnaires to assess pain and fatigue, HQoL, and overall physical function. tDCS was effective in improving pain, fatigue, and physical function and led to a higher mean delta on the mean and maximum pain intensity and FSS.

The strengths of this study were that the patients were screened before tDCS and aerobic exercise training based on pain, fatigue perception, and clinical and anthropometric characteristics. Uni-multidimensional instruments were used by researchers trained prior to the beginning of the study to collect and record data. Finally, pain assessments were conducted through face-to-face interviews to minimize recording bias. In addition, the application of tDCS by trained researchers is a remarkable strategy for reducing the variability in response to treatment in these patients.

From a clinical point of view, patients with IIMs showed a greater effect size on the functional index for myositis exercise, and similar data were observed in the general SPPB score. These results corroborate the clinical trial study of our group, which showed that the application of three sessions of tDCS was safe and promoted significant improvements in physical function and muscle strength. Potential mechanisms involve facilitation of neural networks [12].

Nonetheless, a greater effect on pain and fatigue was observed in tDCS associated with aerobic exercise training than in the sham group associated with aerobic exercise training (Supplementary Figures S3 and S4). Based on these results, one possibility is that the association of tDCS in the primary motor cortex with aerobic exercise training results in greater functional connectivity in the primary motor, premotor, and sensorimotor areas [26, 27]. In this context, data support these results, showing that five sessions of tDCS associated with aerobic exercise training were effective in managing pain in patients with myofascial pain syndrome [28] and fibromyalgia [10].

However, these results corroborate the findings of previous literature [8, 29]. However, there is still great heterogeneity in the studies regarding sample size, experimental design, and blinding data that mostly favors the use of anodic tDCS in M1 (C3 or C4) and cathodic tDCS in the supraorbital region, despite cathodic stimulation in M1 [9, 30].

However, evidence has shown the existence of significant heterogeneity in the response to tDCS, and approximately 30% of the general population responds positively to cathodic stimulation in M1 due to a combination of factors, such as age, sex, level of physical activity, and comorbidities [30]. In this context, the sample in the present study was mainly composed of sedentary patients with multiple comorbidities, which may have contributed to the positive results observed with cathodic stimulation of M1.

The present study had some limitations. The sample size was insufficient; however, owing to the heterogeneity and economic and social factors, it was not possible to recruit a larger number of patients. Second, the study was conducted at a single center, which compromises the external validity of the data. Finally, characterization of the ideal washout time is necessary for future studies as there are no data on the ideal washout time in studies with tDCS.

To date, the present study used an aerobic training program based on the perception of subjective exertion, data that may vary according to the patients' perception of fatigue and pain, in turn, influenced by the responsiveness of tDCS. Thus, future multicenter studies with an established washout period are of fundamental importance, and an aerobic training program with intensity prescribed using gold methodology, such as an ergospirometry test, should be used.

## 6. Conclusions

The findings of the present study reveal new associations between the clinical rehabilitation strategies for these diseases. However, additional studies comparing different anodic tDCS versus cathodic tDCS neural hubs in M1 associated with aerobic training are necessary, as are studies with larger sample sizes, which may contribute to the findings of the present study.

## Figures and Tables

**Table 1:** Data related to anthropometric, demographic, comorbidities, physical activity levels, pharmacological therapy, and other drugs of the patients with systemic autoimmune myopathies.

	tDCS (*n* = 16)	Sham (*n* = 15)	*P* value
Demographic and anthropometric			
Age (years)	52.9 ± 10.0	53.1 ± 9.1	0.952
Female sex (%)	15 (93.7)	14.0 (93.0)	0.943
White ethnicity (%)	8 (50.0)	10.0 (66.6)	0.564
Weight (kg)	77.2 ± 11.7	77.5 ± 10.6	0.921
Body mass index (kg/m^2^)	31.6 ± 5.4	30.8 ± 5.9	0.950
Comorbidities			
Systemic arterial hypertension (%)	7 (43.7)	7 (46.6)	0.185
Dyslipidemia (%)	5 (31.2)	6 (40.0)	0.894
Diabetes mellitus (%)	3 (18.7)	3 (20.0)	0.126
Depression (%)	0	0	>0.999
Physical activity levels			
Low (%)	11 (68.7)	12 (80.0)	0.669
Moderate (%)	3 (18.7)	3 (20.0)	0.464
High (%)	2 (12.5)	0	0.263
Equivalent for the task (weekly)	478 (209-1099)	132 (0.0-378)	0.043
Pharmacological therapy			
Glucocorticoids (%)	6 (37.5)	8 (53.3)	0.600
Daily dosage (mg)	0.0 (0.0-8.0)	5.0 (0.0-17.0)	0.731
IS/IM (%)	10 (62.5)	12 (80.0)	0.498
Leflunomide (%)	3 (18.7)	3 (20.0)	0.126
Methotrexate (%)	5 (31.2)	7 (46.6)	0.608
Azathioprine (%)	2 (12.5)	3 (20.0)	0.932
Mycophenolate of mofetil (%)	6 (37.5)	4 (26.6)	0.810
Rituximab (%)	0	0	>0.999
Other drugs			
Antihypertensive (%)	5 (31.2)	4 (26.6)	0.443
Pain relievers (%)	4 (25.0)	4 (26.6)	0.647
Statins (%)	1 (6.2)	3 (20.0)	0.545
Antidepressants (%)	0	0	>0.999

Data presented as mean ± standard deviation, median (25^th^-75^th^), or frequency (%). IM: immunomodulators; IS: immunosuppressants; tDCS: transcranial direct current stimulation.

**Table 2:** Data related to the clinical characteristics before and after the intervention with tDCS.

Clinical parameters	tDCS (*n* = 16)			Sham (*n* = 15)			*P* ^∗^group	*P* ^∗^time	*P* ^∗^group − time
	Pre	Post	ES	Pre	Post	ES			
Patients' VAS (0-10 cm)	0.0 (0.0-3.0)	1.2 (0.0-2.8)	-0.02	2.0 (0.0-5.5)	0.0 (0.0-0.0)	-0.38	0.842	0.085	0.524
Physicians' VAS (0-10 cm)	0.0 (0.0-0.0)	0.0 (0.0-0.0)	0	0.0 (0.0-0.0)	0.0 (0.0-0.0)	-0.26	0.786	0.926	0.585
MMT-8 (0-80)	80 (80-80)	80 (80-80)	0	80 (80-80)	80 (80-80)	0	0.370	0.031	0.533
HAQ (0-3.00)	0.25 (0.0-0.93)	0.00 (0.00-0.31)	-0.49	0.25 (0.00-0.87)	0.00 (0.00-0.37)	-0.37	0.151	0.042	0.002
FI-3 elbow (0-60)	52.3 ± 15.4	60.8 ± 6.8	0.55	57.0 ± 12.2	59.3 ± 10.6	0.19	0.148	0.189	0.112
F-3 elbow (Borg) (0-10)	8.3 ± 3.1	8.2 ± 2.8	-0.03	8.9 ± 1.4	8.8 ± 2.1	-0.05	0.612	0.171	>0.999
FI-3 cervical (0-60)	49.4 ± 17.8	60.6 ± 8.2	0.78	48.1 ± 21.8	50.3 ± 12.7	0.12	0.048	0.014	0.047
F-3 cervical (Borg) (0-10)	8.9 ± 2.0	8.5 ± 2.8	-0.19	8.7 ± 2.2	8.8 ± 2.0	0.06	0.890	0.701	0.299
FI-3 hip (0-60)	44.0 ± 18.0	57.7 ± 9.5	0.74	50.0 ± 19.2	53.6 ± 15.8	0.19	0.061	0.420	0.003
FI-3 hip (Borg) (0-10)	9.4 ± 1.8	8.7 ± 2.6	-0.40	9.5 ± 1.2	9.3 ± 1.9	-0.11	0.615	0.779	0.680
MYOACT (0-10)	0.0 (0.0-0.0)	0.0 (0.0-0.0)	-0.11	0.0 (0.0-0.0)	0.0 (0.0-0.0)	-0.26	0.633	0.62	0.687
CPK (U/L)	112 (68-200)	156 (92-294)	0.33	110 (68-178)	196 (94-200)	0.41	0.918	0.321	0.497
ALT (U/L)	19 (17-21)	18 (17-19)	-0.40	20 (17-23)	17 (15-18)	-0.57	0.999	0.813	0.953
AST (U/L)	17 (14-22)	16 (16-20)	-0.07	18 (17-23)	18 (17-18)	-0.44	0.995	0.948	0.941

Data are presented as mean, standard deviation, median (25^th^-75^th^), or frequency (%). ALT: alanine aminotransferase; AST: aspartate aminotransferase; CPK: creatine phosphokinase; HAQ: Health Assessment Questionnaire; FI: Functional Index; MMT-8: Manual Muscle Testing-8; MYOACT: Myositis Disease Activity Assessment Visual Analog Scales; tDCS: transcranial direct current stimulation; ES: effect size.

**Table 3:** Data related to the physical function.

	tDCS (*n* = 16)			Sham (*n* = 15)			*P* ^∗^ group	*P* ^∗^ time	*P* ^∗^group − time
	Pre	Post	ES	Pre	Post	ES			
SPPB mobility (s)	3.1 (2.6-3.3)	2.7 (2.5-2.9)	-0.31	3.1 (2.5-3.5)	2.8 (2.4-3.1)	-0.19	0.626	0.919	0.081
SPPB balance (s)	10 (10-10)	10 (10-10)	0.25	10 (10-10)	10 (10-10)	0	0.381	0.276	0.369
SPPB strength (s)	13.2 (10-17.8)	10.3 (9.9-15.1)	-0.49	15 (10.5-16.7)	14.6 (10.5-15.8)	3.30	0.063	0.052	0.001
SPPB score (0-12)	8.6 ± 1.4	9.5 ± 0.8	0.62	8.4 ± 1.4	8.4 ± 1.3	0.05	0.044	0.060	0.042
TUG (s)	7.6 ± 1.1	6.7 ± 0.7	-0.80	7.7 ± 1.3	7.2 ± 0.8	-0.42	0.094	0.064	0.061
TST (reps)	13.1 ± 4.5	15.3 ± 2.6	0.48	12.3 ± 2.7	13.5 ± 3.2	0.43	0.612	0.243	0.054

Data are presented as mean ± standard deviation, or median (25^th^-75^th^). SPPB: Short Physical Performance Battery; tDCS: transcranial direct current stimulation; TUG: timed up and go test; TST: timed stand test; ES: effect size.

**Table 4:** Data related to the quality of life before and after the intervention.

	tDCS (*n* = 16)			Sham (*n* = 15)			*P* ^∗^ group	*P* ^∗^ time	*P* ^∗^group − time
	Pre	Post	ES	Pre	Post	ES			
Physical function	62.5 (48.8-72.5)	70 (60-88)	0.38	55 (40-67.5)	55 (45-63.8)	0.14	0.281	0.770	0.034
Physical aspect	95 (50-100)	100 (68-100)	0.23	50 (0.0-100)	50 (37.5-100)	0.15	0.492	0.267	0.941
Pain	35 (28.8-52.5)	20 (0.0-33.1)	-0.71	30 (10-40)	40 (30-50)	0.48	0.205	0.453	0.928
General health	70 (46.2-81.2)	70 (57.5-75)	0.18	70 (57.5-82.5)	60 (50-77.5)	-0.09	0.943	0.160	0.252
Vitality	52.5 (41.2-6.2)	47.5 (40-60)	-0.12	45 (25-55)	45 (40-60)	0.29	0.59	0.952	0.040
Social aspects	45 (34.8-50)	50 (38-53.2)	0.02	50 (38-62.5)	50 (44-66.5)	0.32	0.422	0.287	0.240
Emotional aspects	100 (33-100)	100 (62.6-100)	0.08	67 (33-100)	67 (16.5-100)	-0.14	0.692	0.748	0.249
Mental health	49 (39-56)	49 (44-60)	0.06	48 (44-60)	50 (40-56)	0.04	0.883	0.176	0.156

Data are presented as median (25^th^-75^th^). tDCS: transcranial direct current stimulation; ES: effect size.

**Table 5:** Data regarding the perception and severity of fatigue before and after the intervention.

	tDCS (*n* = 16)			Sham (*n* = 15)			*P* ^∗^ group	*P* ^∗^ time	*P* ^∗^group − time
	Pre	Post	ES	Pre	Post	ES			
FSS (1-63)	4.1 ± 1.9	2.9 ± 1.6	-0.61	3.7 ± 2.2	3.5 ± 2.1	-0.11	0.361	0.041	0.014
VASf (0-10)	5.0 (2.0-8.2)	2.5 (0.0-5.0)	-0.53	5.0 (2.0-8.0)	3.0 (1.5-5.0)	-0.36	0.221	0.141	0.023

Data are presented as mean ± standard deviation, or median (25^th^-75^th^). VASf: visual analog fatigue scale; FFS: Fatigue Severity Scale; tDCS: transcranial direct current stimulation; ES: effect size.

**Table 6:** Data regarding the perception and impact of pain before and after interventions.

	tDCS (*n* = 16)			Sham (*n* = 15)			*P* ^∗^ group	*P* ^∗^ time	*P* ^∗^group − time
	Pre	Post	ES	Pre	Post	ES			
VASp moment (0-10)	1.5 (0.0-5.0)	0.0 (0.0-0.3)	-0.33	0.0 (0.0-5.5)	4.0 (0.0-5.0)	0.17	0.589	0.025	0.934
VASp minimum (0-10)	2.0 (1.0-3.0)	0.5 (0.0-1.0)	-0.60	3.0 (1.0-5.0)	1.0 (1.0-3.0)	-0.54	0.097	0.353	0.432
VASp average (0-10)	3.7 (2.2-5.5)	0.0 (0.0-2.2)	-0.79	3.0 (1.0-5.0)	2.0 (1.5-5.0)	-0.14	0.097	0.017	0.004
VASp maximum (0-10)	6.0 (4.0-8.2)	1.0 (0.0-4.0)	-1.10	5.0 (1.0-8.0)	4.0 (0.0-6.5)	-0.24	0.016	0.083	0.023
Brief pain impact									
VASp general activity (0-10)	1.0 (0.0-8.2)	0.0 (0.0-3.2)	-0.45	5.0 (1.0-8.5)	3.0 (0.0-5.0)	-0.35	0.251	0.053	0.027
VASp disposition (0-10)	1.0 (0.0-6.2)	0.0 (0.0-1.1)	-0.39	3.0 (0.0-4.5)	3.0 (0.0-5.0)	-0.1	0.748	0.021	0.37
VASp walking ability (0-10)	0.5 (0.0-6.5)	0.0 (0.0-0.0)	-0.49	0.0 (0.0-7.5)	0.0 (0.0-5.0)	-0.16	0.312	0.151	0.039
VASp work (0-10)	0.0 (0.0-8.0)	0.0 (0.0-0.5)	-0.50	4.0 (0.0-6.0)	0.0 (0.0-5.0)	-0.29	0.293	0.297	0.002
VASp interpersonal relationship (0-10)	0.0 (0.0-4.0)	0.0 (0.0-0.0)	-0.31	0.0 (0.0-6.0)	0.0 (0.0-2.5)	-0.29	0.381	0.211	0.089
VASp sleep (0-10)	0.0 (0.0-4.2)	0.0 (0.0-3.1)	-0.10	0.0 (0.0-7.0)	0.0 (0.0-4.0)	-0.24	0.787	0.571	0.063
VASp enjoyment of life (0-10)	0.0 (0.0-6.5)	0.0 (0.0-0.5)	-0.40	0.0 (0.0-6.0)	0.0 (0.0-3.5)	-0.17	0.408	0.073	0.029
McGill score (0-84)	32 (1.5-38.0)	0.0 (0.0-13.4)	-0.78	26 (0.0-37)	5.0 (0.0-19.2)	-0.54	0.047	0.058	0.447

Data are presented as median (25^th^-75^th^). VASp: visual pain scale; tDCS: transcranial direct current stimulation.

## Data Availability

Data is available in the Supplementary Information files. Additional information is also available on request to the corresponding author.

## Supplementary Materials

Supplementary 1 Table S1: data regarding patient blinding and adverse events. Legends: data presented as frequency (%). tDCS: transcranial direct current stimulation.

Supplementary 2 Figure S1: study flowchart. Legends: tDCS: transcranial direct current stimulation; AE: aerobic exercise training.

Supplementary 3 Figure S2: randomization. Legends: tDCS+AE: transcranial direct current stimulation; Sham+AE: sham stimulation-associated aerobic exercise training.

Supplementary 4 Figure S3: fatigue severity data (A and B). Legends: FSS: Fatigue Severity Scale; tDCS: transcranial direct current stimulation. Mean Δ : mean of (post − pre/pre) × 100.

Supplementary 5 Figure S4: individual data referring to a minimum (A and B), average (C and D), and maximum (E and F) perception of pain of patients with systemic autoimmune myopathies undergoing intervention with tDCS or placebo. Legends: VASp: visual analog scale for pain; tDCS: transcranial direct current stimulation. Mean Δ : mean of (post − pre/pre) × 100.
