# Supplementary material for: Impact of Transcranial Direct Current Stimulation in Pain, Fatigue, and Health Quality of Life of Patients with Idiopathic Inflammatory Myopathies: A Randomized, Double-Blind, Sham-Controlled Crossover Clinical Trial
Source: Int J Rheumatol. 2024 Feb 1;2024:1583506. doi: 10.1155/2024/1583506 (PMC10853024; doi:10.1155/2024/1583506)
Supplement: Supplementary 1 — Table S1: data regarding patient blinding and adverse events. Legends: data presented as frequency (%). tDCS: transcranial direct current stimulation. [file 1583506.f1.docx]

| **Supplementary materials**  **Table S1**. Data regarding patient blinding and adverse events | | | | |
| --- | --- | --- | --- | --- |
|  | Post 1^st^ Intervention | | Post 2^st^ Intervention | |
|  | tDCS (n=8) | Sham (n=6) | tDCS (n=8) | Sham (n=6) |
| Believe it stimulated |  |  |  |  |
| Yes | 5 (62.5) | 8 (88.8) | 4 (50.0) | 3 (50.0) |
| No | 3 (37.5) | 1 (11.1) | 4 (50.0) | 3 (50.0) |
| Adverse effects |  |  |  |  |
| Yes | 4 (50.0) | 2 (22.2) | 1 (12.5) | 1 (16.6) |
| No | 4 (50.0) | 7 (77.7) | 6 (75.0) | 4 (66.6) |
| Burning | 2 (25.0) | 1 (11.1) | 0 | 0 |
| Fatigue | 2 (25.0) | 1 (11.1) | 1 (12.5) | 1 (16.6) |
| General status change | 0 | 0 | 0 | 0 |

Data presented as frequency (%). tDCS: transcranial direct current stimulation.
