# Supplementary figures and images for: Impact of Transcranial Direct Current Stimulation in Pain, Fatigue, and Health Quality of Life of Patients with Idiopathic Inflammatory Myopathies: A Randomized, Double-Blind, Sham-Controlled Crossover Clinical Trial
Source: Int J Rheumatol. 2024 Feb 1;2024:1583506. doi: 10.1155/2024/1583506 (PMC10853024; doi:10.1155/2024/1583506)

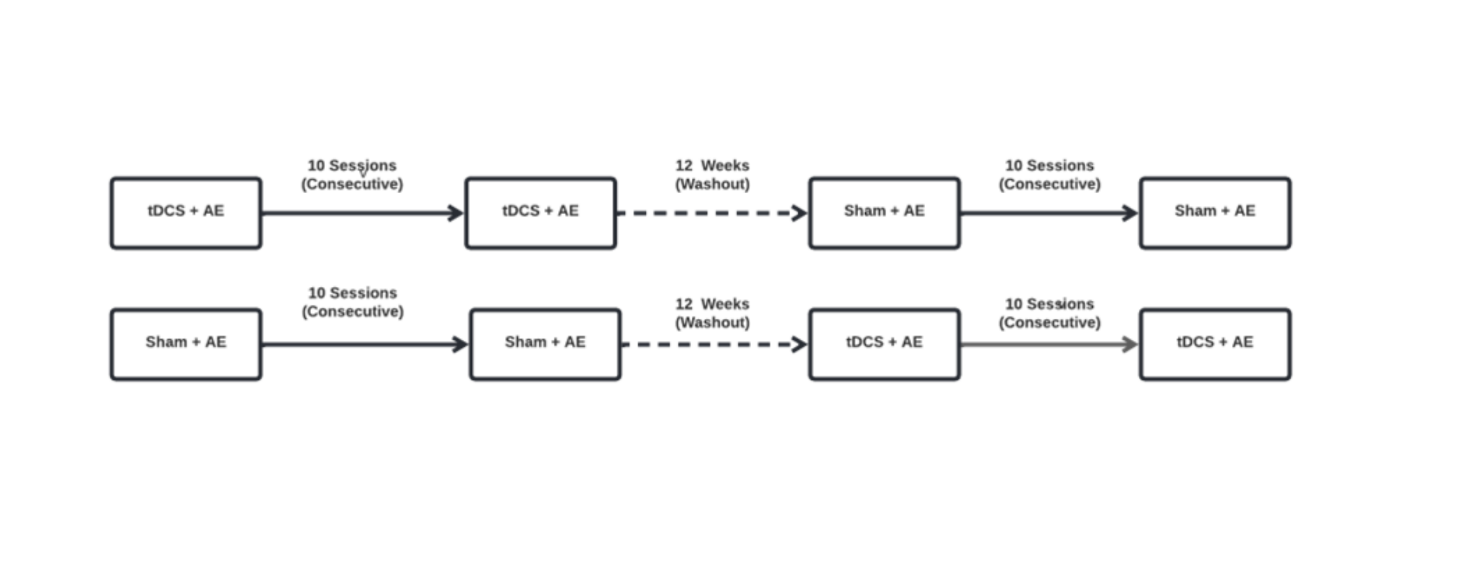

Supplement: Supplementary 2 — Figure S1: study flowchart. Legends: tDCS: transcranial direct current stimulation; AE: aerobic exercise training. [file 1583506.f2.docx]

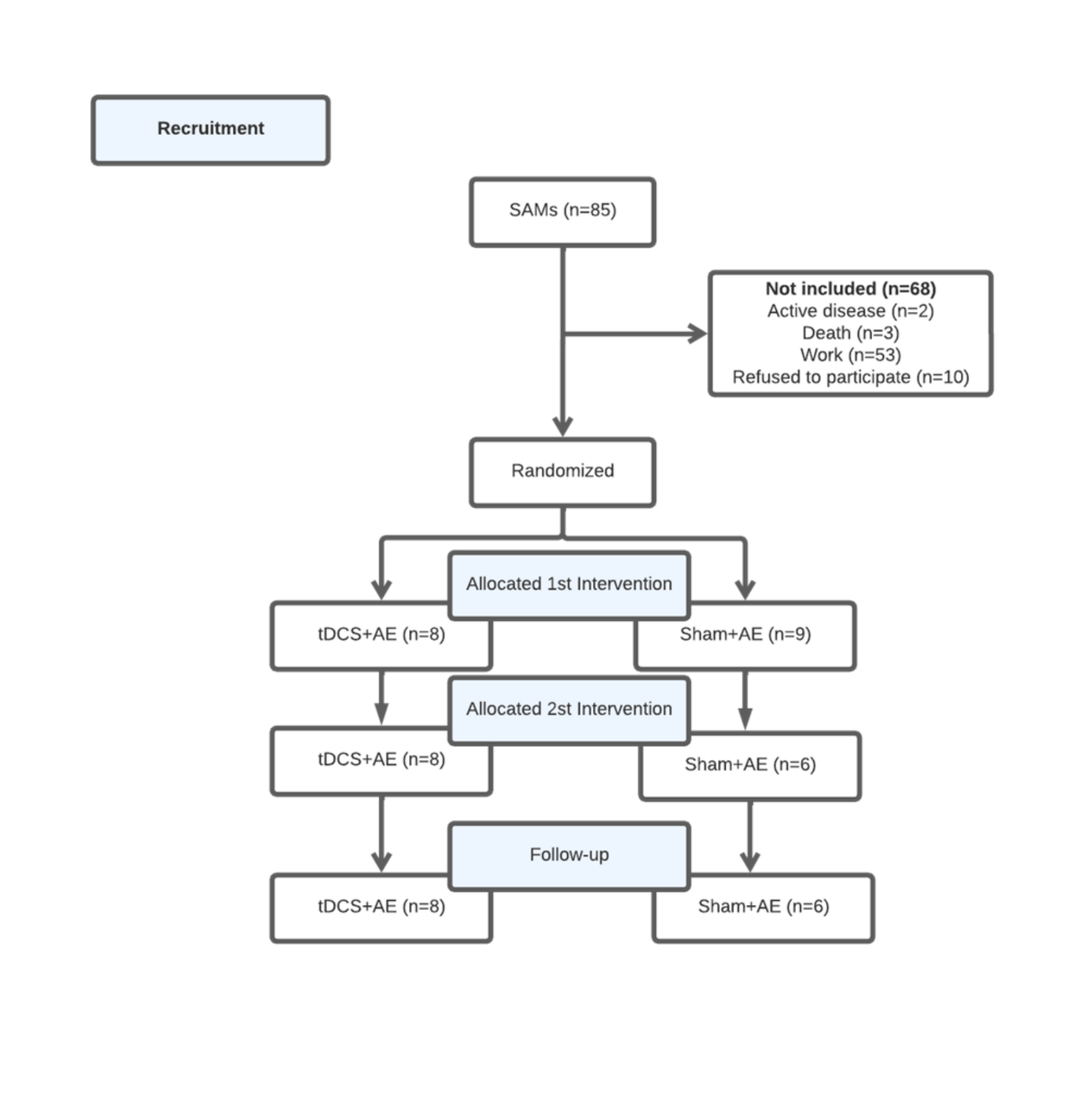

Supplement: Supplementary 3 — Figure S2: randomization. Legends: tDCS+AE: transcranial direct current stimulation; Sham+AE: sham stimulation-associated aerobic exercise training. [file 1583506.f3.docx]

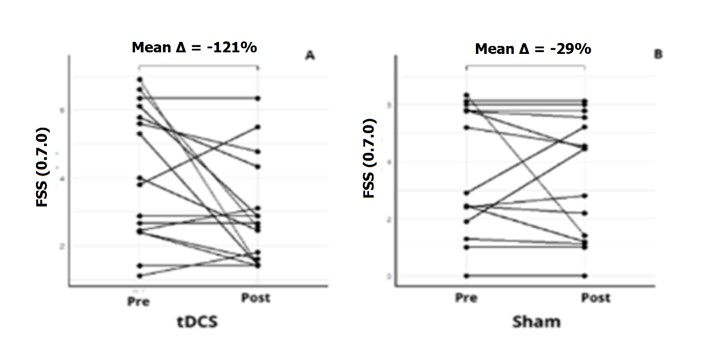

Supplement: Supplementary 4 — Figure S3: fatigue severity data (A and B). Legends: FSS: Fatigue Severity Scale; tDCS: transcranial direct current stimulation. Mean Δ : mean of (post − pre/pre) × 100. [file 1583506.f4.docx]

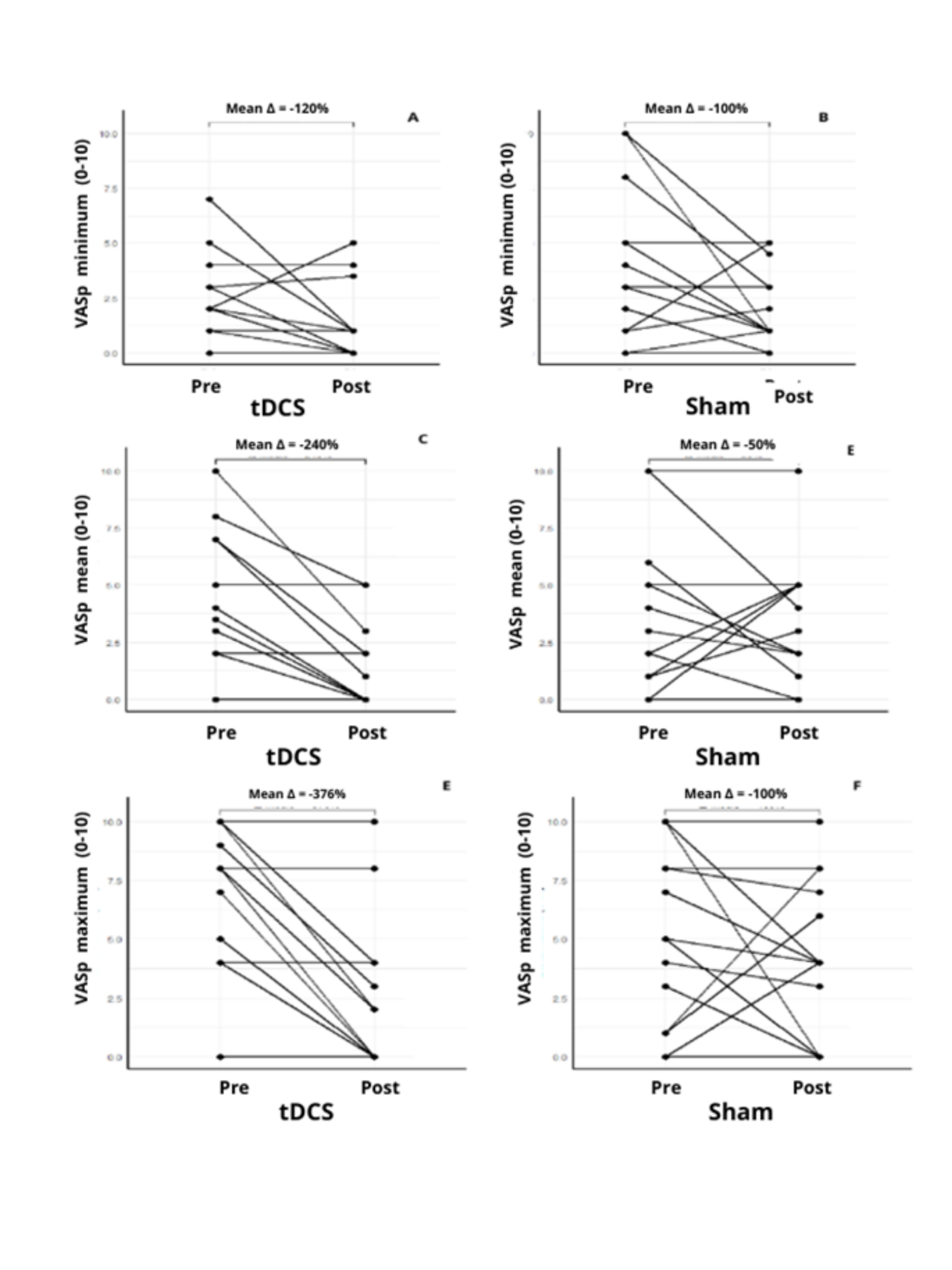

Supplement: Supplementary 5 — Figure S4: individual data referring to a minimum (A and B), average (C and D), and maximum (E and F) perception of pain of patients with systemic autoimmune myopathies undergoing intervention with tDCS or placebo. Legends: VASp: visual analog scale for pain; tDCS: transcranial direct current stimulation. Mean Δ : mean of (post − pre/pre) × 100. [file 1583506.f5.docx]
